# Triclinic polymorph of bis­[2-methyl-3-(pyridin-2-yl)imidazo[1,5-*a*]pyridin-2-ium] tetra­chloridocadmium(II)

**DOI:** 10.1107/S2056989024009654

**Published:** 2024-10-04

**Authors:** Olga Yu. Vassilyeva, Elena A. Buvaylo, Vladimir N. Kokozay, Evgeny A. Goreshnik

**Affiliations:** a Department of Chemistry, Taras Shevchenko National University of Kyiv, Volodymyrska str. 64/13, 01601 Kyiv, Ukraine; b Department of Inorganic Chemistry and Technology, Jožef Stefan Institute, Jamova 39, 1000 Ljubljana, Slovenia; Illinois State University, USA

**Keywords:** crystal structure, Cd^II^, organic–inorganic hybrid, tetra­halometallate, π–π stacking, photoluminescence.

## Abstract

The crystal structure of the title organic–inorganic hybrid salt, (C_13_H_12_N_3_)_2_[CdCl_4_], (I), has been reported with four mol­ecules in the asymmetric unit in a monoclinic cell [Vassilyeva *et al.* (2021 ▸). *RSC Advances*, **11**, 7713–7722]. While using two different aldehydes in the oxidative cyclization–condensation involving CH_3_NH_2_·HCl to prepare a new monovalent cation with the imidazo[1,5-*a*]pyridinium skeleton, a new polymorph was obtained for (I) in space group *P*1 and a unit cell with approximately half the volume of the monoclinic form. The structural configurations of the two crystallographically non-equivalent organic cations as well as the geometry of the moderately distorted tetra­hedral CdCl_4_^2–^ dianion show minor changes. In the crystal, identically stacked cations and tetra­chloro­cadmate anions form separate columns parallel to the *a* axis. The loose packing of the anions leads to a minimal separation of approximately 9.53 Å between the metal atoms in the triclinic form *versus* 7.51 Å in the monoclinic one, indicating that the latter is packed slightly more densely. The two forms also differ by aromatic stacking motifs. Similar to the monoclinic polymorph, the triclinic one excited at 364 nm shows an intense unsymmetrical photoluminescent band with maximum at 403 nm and a full width at half maximum of 51 nm in the solid state.

## Abstract 2

The photoluminescent properties of the monoclinic and triclinic polymorphs in the solid state are strictly comparable, presumably because the spatial organization of both polymorphs is quite similar.

## 1. Chemical context

Polymorphism – the existence of more than one crystal structure for a given material – is of inter­est in many research areas and applications ranging from crystallography and solid-state chemistry, materials science, and pharmaceuticals, to agricultural chemistry and food industry. Understanding differences in polymorphs properties is essential for selecting the right form for specific applications, optimizing material performance, and providing better predictive models for crystal formation (Bergeron *et al.*, 2021 ▸; Cai *et al.*, 2023 ▸; Cruz-Cabeza *et al.*, 2020 ▸). The control of the mol­ecular assemblies during the crystallization process of a polymorphic cyclo­metalated Ir^III^ ethyl­enedi­amine complex was demonstrated as an efficient tool to modulate emission and limit the aggregation-quenching phenomenum in the solid crystalline state (Talarico *et al.*, 2010 ▸). The inter­molecular inter­actions in two polymorphic modifications of a platinum emitter with 3-(benzen-2-id­yl)-1-methyl-1,3-di­hydro-2*H*-imidazo[4,5-*b*]pyridin-2-yl­idene ligand have been shown to strongly affect its photophysical properties and even make the polymorphs separable (Pinter *et al.*, 2021 ▸).

In a previous study, we used organic–inorganic hybrid salts made of imidazo[1,5-*a*]pyridinium-based cations and tetra­chloro­cadmate anions as fluorescent agents to modify cross-linked polyurethane (CPU; Vassilyeva *et al.*, 2021 ▸). The use of ionic compounds immobilized *in situ* in the CPU in low content (1 wt%) ensured excellent dispersion of components in the polymer matrix so that uniformly luminescent films were fabricated. In [*L*]_2_[CdCl_4_], 2-methyl-3-(pyridin-2-yl)imidazo[1,5-*a*]pyridinium cations (*L*^+^) resulted from the oxidative cyclization–condensation involving CH_3_NH_2_·HCl and 2-pyridine­carbaldehyde (2-PCA) in methanol. The developed synthetic approach enables the systematic modification of the photoluminescent properties of imidazo[1,5-*a*]pyridine species through varying substituents on the polyheterocyclic core as well as through introduction of different metal ions (Vassilyeva *et al.*, 2020 ▸). In the present work, an attempt to prepare another electron-deficient monovalent cation with the imidazo[1,5-*a*]pyridinium skeleton by replacing half the amount of 2-PCA with 2-hy­droxy-3-meth­oxy­benzaldehyde in the reaction with CH_3_NH_2_·HCl appeared unsuccessful due to presumably insufficient reaction time. The isolated compound was crystallographically identified as a new triclinic polymorph of [*L*]_2_[CdCl_4_], (I), which was reported previously in space group *P*2_1_/*c* [Cambridge Structural Database (CSD) refcode GOSYUL; Vassilyeva *et al.*, 2021 ▸]. The photoluminescent properties of the monoclinic and triclinic polymorphs in the solid state were found to be very similar, suggesting that structural differences of the two modifications of the organic–inorganic hybrid material are not significant enough to affect their photophysical properties.
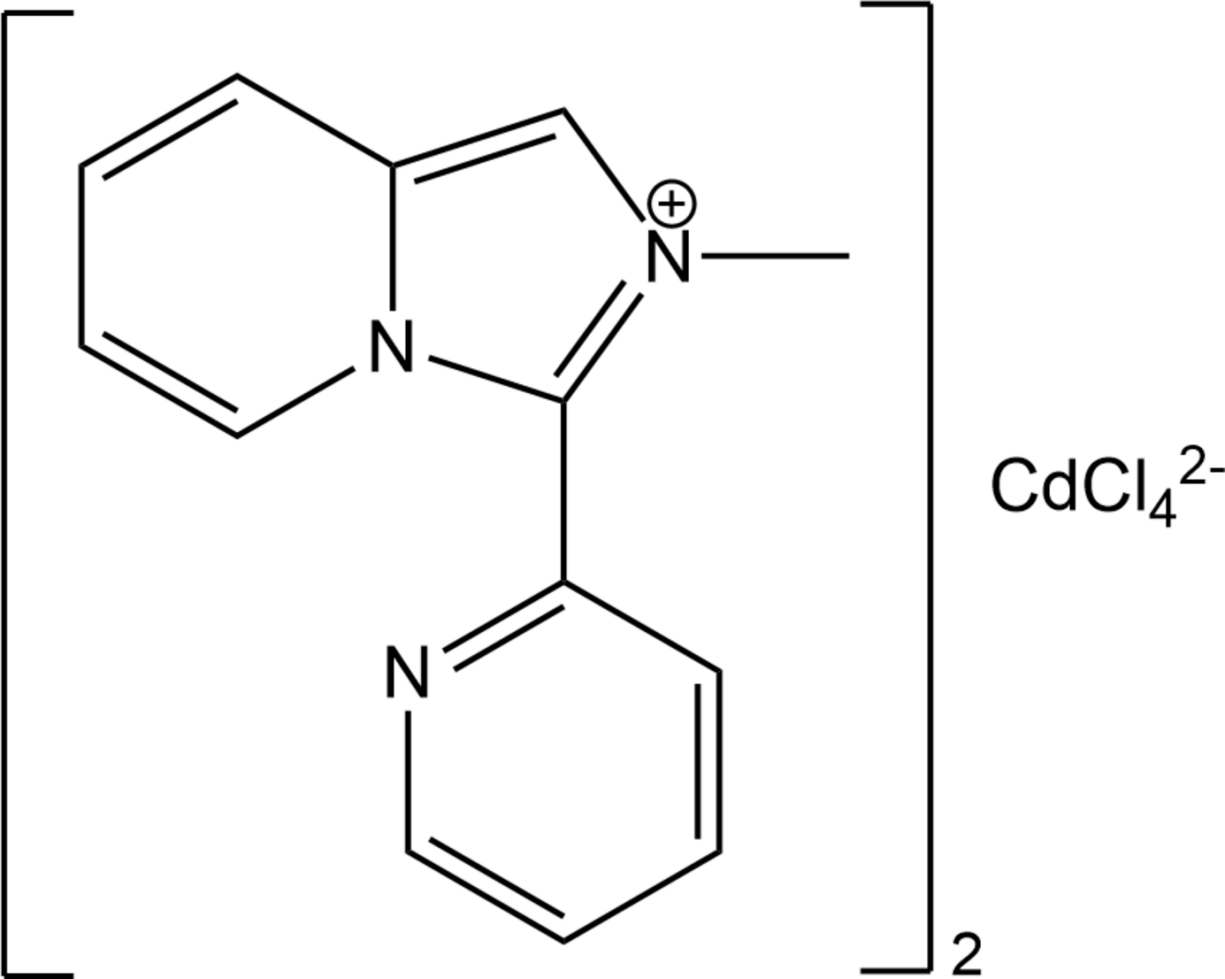


## 2. Structural commentary

**Figure 1:**
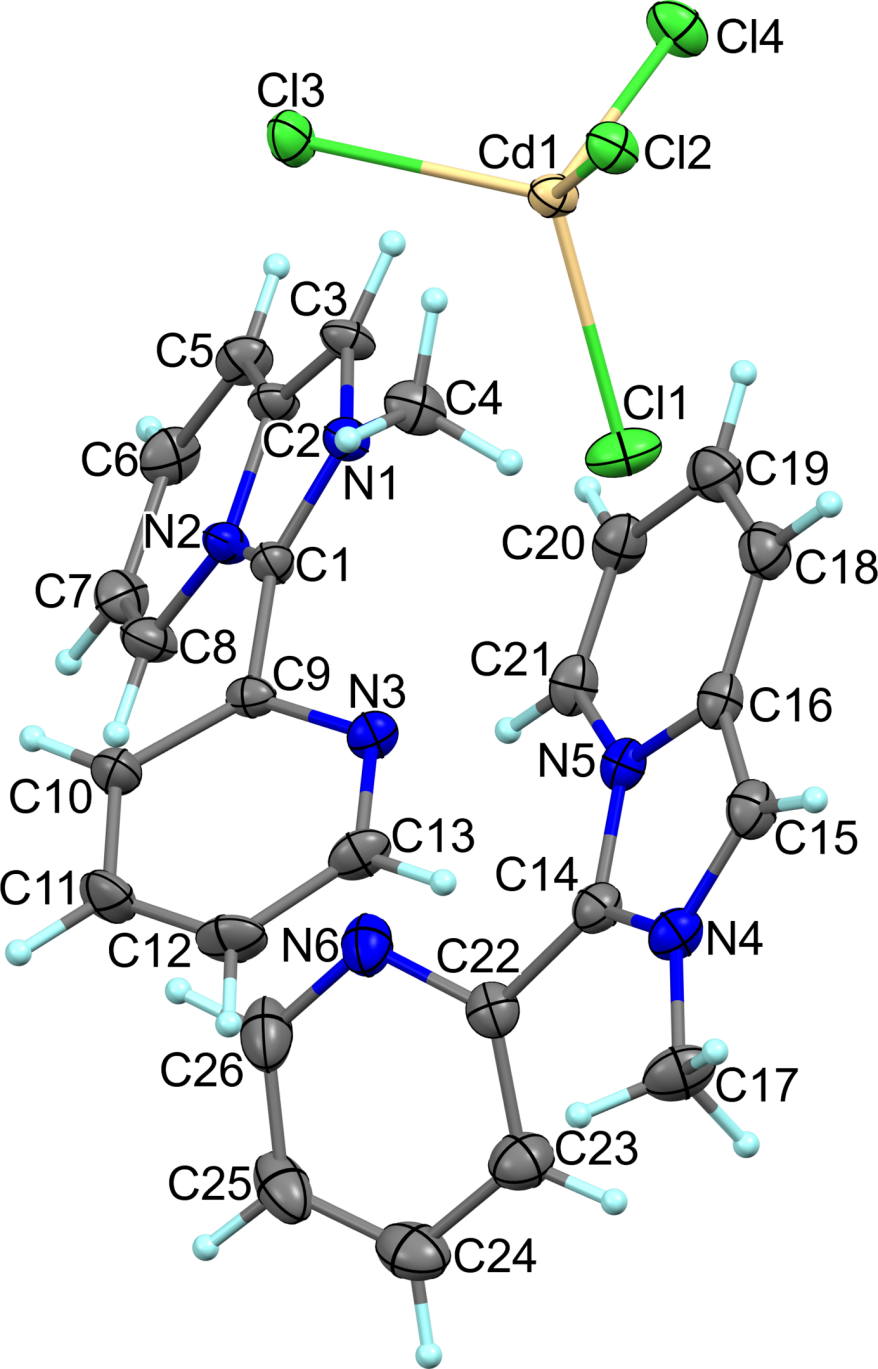
Mol­ecular structure and labelling of the triclinic polymorph of [*L*]_2_[CdCl_4_] with ellipsoids at the 50% probability level.

Triclinic crystals of [*L*]_2_[CdCl_4_], which crystallize in the space group *P*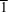, contain discrete organic cations and tetra­chloro­cadmate anions (Fig. 1 ▸). The structural configurations of two crystallographically non-equivalent *L*1(N1, N2) and *L*2(N4, N5) cations are similar with small differences in bond distances and angles. The pyridinium rings in the flattened fused cores have expected bond lengths; the bond distances in the imidazolium entities are in the range 1.341 (3)–1.399 (3) Å. The five- and six-membered rings in the cores are almost coplanar showing dihedral angles between them of approximately 2.46 (*L*1) and 2.08° (*L*2). The geometric parameters of the cations are highly comparable to those found in monoclinic GOSYUL except for the dihedral angles between the pendant pyridyl rings and the planes of the remainder of the cation. The dihedral angles are about 43.35 and 40.04° for *L*1 and *L*2, respectively, in (I) and 36.4 (2), and 35.9 (2)° for the two crystallographically non-equivalent cations in GOSYUL. The tetra­hedral CdCl_4_^2–^ anion is more distorted compared with the anion geometry in the monoclinic polymorph. The Cd—Cl distances vary from 2.4436 (7) to 2.4895 (6) Å and the Cl—Cd—Cl angles fall in the range 100.36 (2)–115.56 (2)° (Table 1 ▸). The maximum differences in the lengths and angles are 0.0459 (7) Å and 15.2 (2)°, respectively, while those in GOSYUL amount to 0.048 Å and 4.94°.

## 3. Supra­molecular features

**Figure 2:**
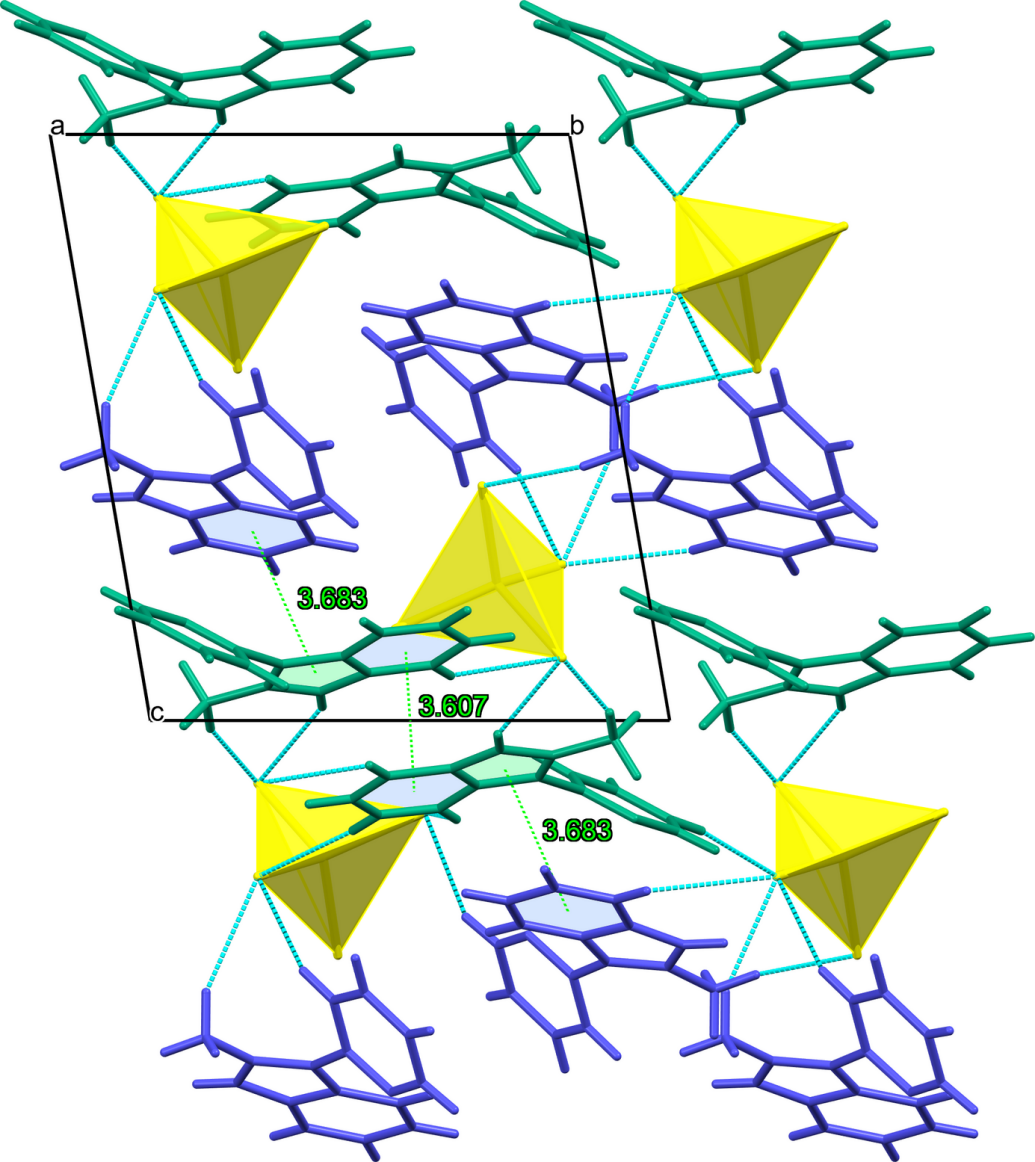
Fragment of the crystal packing of the triclinic polymorph of [*L*]_2_[CdCl_4_] viewed along the *a* axis and showing the *L*1 and *L*2 independent cations (green and blue) joined by aromatic stacking and the inter­molecular C—H⋯Cl contacts given as blue dashed lines.

In the crystal, identically stacked *L*1, *L*2 cations and CdCl_4_^2–^ anions form separate columns parallel to the *a* axis (Fig. 2 ▸). The cations from neighbouring columns are involved in aromatic stacking between the offset pyridinium and imidazolium entities of the fused cores with ring–centroid distances of 3.607 (1) and 3.683 (1) Å. The π–π stacking between the adjacent pendant pyridyl rings of *L*1 and *L*2, which are twisted to each other by approximately 43.81°, is negligible [the ring-centroid separation is 4.344 (1) Å]. The loose packing of the tetra­chloro­cadmate anions leads to a closest separation of approximately 9.53 Å between the metal atoms in the crystal. This separation in GOSYUL is equal to 7.51 Å, indicating that the latter is packed slightly more densely, while the spatial organization of both polymorphs remains rather similar (Figs. 2 ▸, 3 ▸). The title compound lacks classical hydrogen-bonding inter­actions. Additional structure consolidation is provided by several C—H⋯Cl—Cd hydrogen bonds between organic and inorganic counterparts (Table 2 ▸) at H⋯Cl distances below the van der Waals contact limit of 2.85 Å (Mantina *et al.*, 2009 ▸). The longer contacts are considered a result of the crystal packing.

## 4. Database survey

More than 50 structures of salts with imidazo[1,5-*a*]pyridin­ium-based cations have been deposited in the CSD (Version 5.45, update March 2024; Groom *et al.*, 2016 ▸) with above half of them being a contribution from our research group. These span Mn, Co, Fe, Ni, Cu, Zn, Cd, Pb and Sn halometalates (Cl, Br, I) with 2-methyl­imidazo[1,5-*a*]pyridin-2-ium, 2-methyl-3-(pyridin-2-yl)imidazo[1,5-*a*]pyridin-2-ium (*L*^+^), 2-hy­droxy­ethyl­imidazo[1,5-*a*]pyridin-2-ium and 2,2′-(ethane-1,2-di­yl)bis­(imidazo[1,5-*a*]pyridin-2-ium) cations. Most of them either are built of cations and anions arranged in separate columns similar to (I) or show pseudo-layered structures with alternating sheets of organic cations and of halometalate anions (Buvaylo *et al.*, 2015 ▸; Vassilyeva *et al.*, 2019 ▸, 2021 ▸). Lead halide hybrid perovskites from the series are distinguished by their one-dimensional architecture (Vassilyeva *et al.*, 2020 ▸, 2023 ▸).

Organic salts with imidazo[1,5-*a*]pyridinium cations having varying substituents on the imidazolium ring and inorganic anions such as hexa­fluoro­phosphate or perchlorate constitute another large group. The structures similar to (I) are 2-(2-pyrid­yl)-*N*^3^-(4-chloro­phen­yl)imidazo[1,5-*a*]pyridinium per­chlorate (YIHFEB; Mitra *et al.*, 2007 ▸) and 2-(2-(1*H*-imidazol-3-ium-5-yl)eth­yl)-3-(pyridin-2-yl)imidazo[1,5-*a*]pyridin-2-ium diperchlorate (UREYIA; Türkyılmaz *et al.*, 2011 ▸) with chloro­phenyl and ethyl­imidazolium substituents in place of the methyl group in *L*^+^, respectively. 3-(Pyridin-2-yl)imidazo[1,5-*a*]pyridine, a neutral *L* mol­ecule lacking the methyl group, was reported to crystallize in ortho­rhom­bic space group *P*2_1_2_1_2_1_ (PRIMPY; Golic *et al.*, 1980 ▸). It acts as a *κ*^2^(*N*,*N*) chelator to form an Mn^II^ complex (Álvarez *et al.*, 2012 ▸) but can be easily released from the complex by boiling its suspension in water.

The ubiquitous Cd tetra­chloride anion is found in more than 300 CSD structures. The mean Cd—Cl bond length of 2.46 Å in (I) is comparable to distances found in the database for other salts containing isolated CdCl_4_^2–^ tetra­hedral anions (Cd—Cl distances for this anion vary from 2.38 to 2.57 Å with the average lengths falling in the narrow range 2.43–2.48 Å).

## 5. Photoluminescence measurements

**Figure 4:**
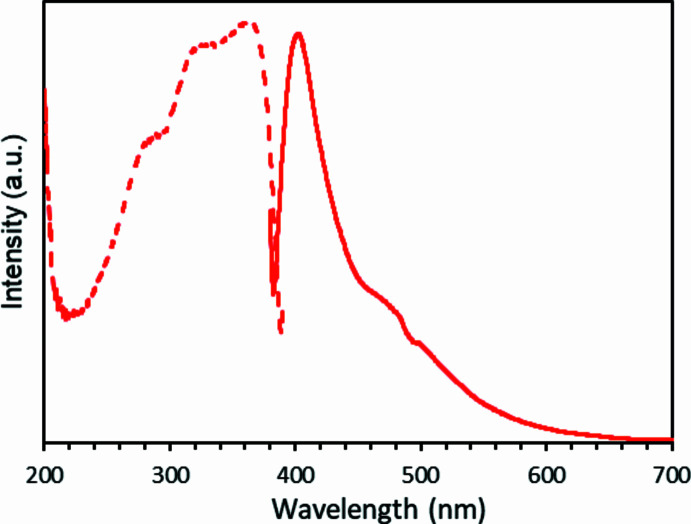
The excitation (dotted) and emission spectra (solid) of a powdered sample of the triclinic polymorph of [*L*]_2_[CdCl_4_] at room temperature.

The photoluminescence spectrum of the crystalline powder sample of (I) excited at 364 nm (spectro­fluoro­photometer RF-6000, Shimadzu) shows a broad intense unsymmetrical band with maximum at 403 nm and a full width at half maximum of 51 nm (Fig. 4 ▸). The spectroscopic data are strictly comparable to those of the monoclinic form of (I) (Vassilyeva *et al.*, 2021 ▸), indicating that the structural variations of the two polymorphs of (I) are insufficient to result in different photophysical properties.

## 6. Synthesis and crystallization

2-PCA (0.19 ml, 2.0 mmol) was added dropwise to CH_3_NH_2_·HCl (0.27 g, 4.0 mmol) and 2-hy­droxy-3-meth­oxy­benzaldehyde (0.30 g, 2.0 mmol) dissolved in 10 ml of methanol in a 50 ml conical flask. The solution was stirred magnetically for half an hour at 323 K. Then, solid CdCl_2_·2.5H_2_O (0.23 g, 1.0 mmol) was added to the flask and the reaction mixture was stirred for another half an hour at 323 K, filtered and left to evaporate. Colourless shiny blocks of (I) suitable for X-ray crystallography formed in several hours. The crystals were filtered off, washed with diethyl ether and dried in air. Yield: 0.16 g, 23% (based on cadmium). ^1^H NMR (400 MHz, DMSO-*d*_6_): δ (ppm) 8.93 (*d*, 1H, *J* = 3.9 Hz, H3), 8.70 (*d*, 1H, *J* = 9.3 Hz, H8), 8.56 (*s*, 1H, H3), 8.25–8.19 (*m*, 2H, H10+H11), 8.03 (*d*, 1H, *J* = 9.3 Hz, H5), 7.76–7.73 (*m*, 1H, H2), 7.37 (*t*, 1H, *J* = 8.1 Hz, H6), 7.23 (*t*, 1H, *J* = 7.1 Hz, H7), 4.31 (*s*, 3H, CH_3_). Analysis calculated for C_26_H_24_Cl_4_N_6_Cd (674.73): C 46.28; H 3.59; N 12.46%. Found: C 45.78; H 3.68; N 12.28%.

## 7. Refinement

Crystal data, data collection and structure refinement details are summarized in Table 3 ▸. Anisotropic displacement parameters were employed for the non-hydrogen atoms. All hydrogen atoms were added at calculated positions and refined by use of a riding model with isotropic displacement parameters based on those of the parent atom (C—H = 0.95 Å, *U*_iso_(H) = 1.2*U*_eq_C for CH, C—H = 0.98 Å, *U*_iso_(H) = 1.5*U*_eq_C for CH_3_). Idealized methyl groups were refined as rotating groups.

## Supplementary Material

Crystal structure: contains datablock(s) I. DOI: 10.1107/S2056989024009654/ej2007sup1.cif

Structure factors: contains datablock(s) I. DOI: 10.1107/S2056989024009654/ej2007Isup2.hkl

CCDC reference: 2388010

Additional supporting information:  crystallographic information; 3D view; checkCIF report

## Figures and Tables

**Figure 3:**
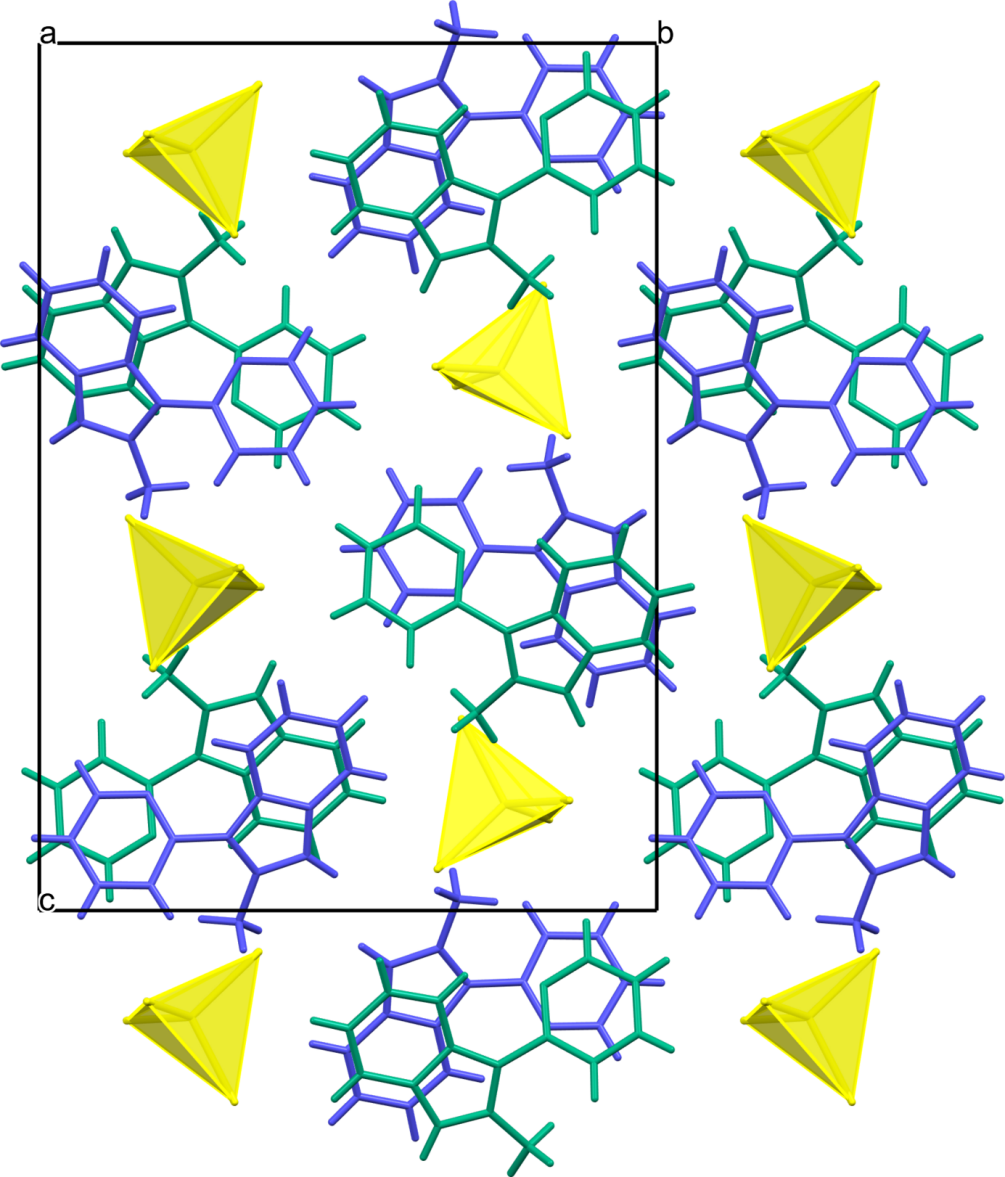
Fragment of the crystal packing of the monoclinic polymorph of [*L*]_2_[CdCl_4_], GOSYUL, viewed along the *a* axis with the independent cations shown in green and blue.

**Table 1:** Selected geometric parameters (Å, °)

Cd1—Cl1	2.4436 (7)	Cd1—Cl3	2.4496 (7)
Cd1—Cl2	2.4448 (6)	Cd1—Cl4	2.4895 (6)
			
Cl1—Cd1—Cl2	108.30 (2)	Cl2—Cd1—Cl3	110.40 (2)
Cl1—Cd1—Cl3	113.98 (2)	Cl2—Cd1—Cl4	115.56 (2)
Cl1—Cd1—Cl4	108.24 (2)	Cl3—Cd1—Cl4	100.36 (2)

**Table 2:** Hydrogen-bond geometry (Å, °)

*D*—H⋯*A*	*D*—H	H⋯*A*	*D*⋯*A*	*D*—H⋯*A*
C4—H4*C*⋯Cl3^i^	0.98	2.72	3.664 (3)	162
C5—H5⋯Cl3	0.95	2.71	3.446 (2)	135
C7—H7⋯Cl4^ii^	0.95	2.72	3.651 (3)	166
C8—H8⋯Cl2^ii^	0.95	2.75	3.518 (3)	138
C12—H12⋯Cl4^iii^	0.95	2.70	3.644 (2)	171
C17—H17*A*⋯Cl4^iv^	0.98	2.79	3.695 (3)	155
C17—H17*C*⋯Cl1^v^	0.98	2.80	3.721 (3)	158
C23—H23⋯Cl4^iv^	0.95	2.69	3.576 (3)	156
C26—H26⋯Cl2^ii^	0.95	2.84	3.689 (3)	149

Symmetry codes: (i) 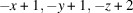; (ii) 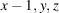; (iii) 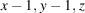; (iv) 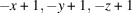; (v) 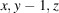.

**Table 3:** Experimental details

Crystal data	
Chemical formula	(C_13_H_12_N_3_)_2_[CdCl_4_]
*M*_r_	674.71
Crystal system, space group	Triclinic, *P* 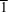
Temperature (K)	150
*a*, *b*, *c* (Å)	9.6288 (3), 11.5103 (4), 13.0302 (5)
α, β, γ (°)	78.734 (3), 81.153 (3), 77.708 (3)
*V* (Å^3^)	1374.33 (9)
*Z*	2
Radiation type	Mo *K*α
μ (mm^−1^)	1.21
Crystal size (mm)	0.44 × 0.27 × 0.20

Data collection	
Diffractometer	New Gemini, Dual, Cu at home/near, Atlas
Absorption correction	Analytical (*CrysAlis PRO*; Rigaku OD, 2023 ▸)
*T*_min_, *T*_max_	0.713, 0.852
No. of measured, independent and observed [*I* > 2σ(*I*)] reflections	23842, 6206, 5235
*R*_int_	0.050
(sin θ/λ)_max_ (Å^−1^)	0.679

Refinement	
*R*[*F*^2^ > 2σ(*F*^2^)], *wR*(*F*^2^), *S*	0.031, 0.065, 1.09
No. of reflections	6206
No. of parameters	336
H-atom treatment	H-atom parameters constrained
Δρ_max_, Δρ_min_ (e Å^−3^)	0.67, −0.54

Computer programs: *CrysAlis PRO* (Rigaku OD, 2023 ▸), *SHELXT2014/4* (Sheldrick, 2015*a* ▸), *SHELXL2016/6* (Sheldrick, 2015*b* ▸), *Mercury* (Macrae *et al.*, 2020 ▸) and *OLEX2* (Dolomanov *et al.*, 2009 ▸).

## supplementary crystallographic information

### Bis[2-methyl-3-(pyridin-2-yl)imidazo[1,5-a]pyridin-2-ium] tetrachloridocadmium(II) Crystal data

**Table:**

(C_13_H_12_N_3_)_2_[CdCl_4_]	*Z* = 2
*M**_r_* = 674.71	*F*(000) = 676
Triclinic, *P*1	*D*_x_ = 1.630 Mg m^−^^3^
*a* = 9.6288 (3) Å	Mo *K*α radiation, λ = 0.71073 Å
*b* = 11.5103 (4) Å	Cell parameters from 12802 reflections
*c* = 13.0302 (5) Å	θ = 2.5–28.5°
α = 78.734 (3)°	µ = 1.21 mm^−^^1^
β = 81.153 (3)°	*T* = 150 K
γ = 77.708 (3)°	Irregular, yellow
*V* = 1374.33 (9) Å^3^	0.44 × 0.27 × 0.20 mm

### Bis[2-methyl-3-(pyridin-2-yl)imidazo[1,5-a]pyridin-2-ium] tetrachloridocadmium(II) Data collection

**Table:**

New Gemini, Dual, Cu at home/near, Atlas diffractometer	6206 independent reflections
Radiation source: fine-focus sealed X-ray tube, Enhance (Mo) X-ray Source	5235 reflections with *I* > 2σ(*I*)
Graphite monochromator	*R*_int_ = 0.050
Detector resolution: 10.6426 pixels mm^-1^	θ_max_ = 28.9°, θ_min_ = 2.2°
ω scans	*h* = −13→12
Absorption correction: analytical (CrysAlisPro; Rigaku OD, 2023)	*k* = −14→15
*T*_min_ = 0.713, *T*_max_ = 0.852	*l* = −17→16
23842 measured reflections	

### Bis[2-methyl-3-(pyridin-2-yl)imidazo[1,5-a]pyridin-2-ium] tetrachloridocadmium(II) Refinement

**Table:**

Refinement on *F*^2^	Primary atom site location: dual
Least-squares matrix: full	Hydrogen site location: inferred from neighbouring sites
*R*[*F*^2^ > 2σ(*F*^2^)] = 0.031	H-atom parameters constrained
*wR*(*F*^2^) = 0.065	*w* = 1/[σ^2^(*F*_o_^2^) + (0.0175*P*)^2^ + 0.519*P*] where *P* = (*F*_o_^2^ + 2*F*_c_^2^)/3
*S* = 1.09	(Δ/σ)_max_ = 0.001
6206 reflections	Δρ_max_ = 0.67 e Å^−^^3^
336 parameters	Δρ_min_ = −0.54 e Å^−^^3^
0 restraints	

### Bis[2-methyl-3-(pyridin-2-yl)imidazo[1,5-a]pyridin-2-ium] tetrachloridocadmium(II) Special details

**Table:**

Geometry. All esds (except the esd in the dihedral angle between two l.s. planes) are estimated using the full covariance matrix. The cell esds are taken into account individually in the estimation of esds in distances, angles and torsion angles; correlations between esds in cell parameters are only used when they are defined by crystal symmetry. An approximate (isotropic) treatment of cell esds is used for estimating esds involving l.s. planes.
Refinement. 1. Fixed Uiso At 1.2 times of: All C(H) groups At 1.5 times of: All C(H,H,H) groups 2.a Aromatic/amide H refined with riding coordinates: C3(H3), C5(H5), C6(H6), C7(H7), C8(H8), C10(H10), C11(H11), C12(H12), C13(H13), C15(H15), C18(H18), C19(H19), C20(H20), C21(H21), C23(H23), C24(H24), C25(H25), C26(H26) 2.b Idealised Me refined as rotating group: C4(H4A,H4B,H4C), C17(H17A,H17B,H17C)

### Bis[2-methyl-3-(pyridin-2-yl)imidazo[1,5-a]pyridin-2-ium] tetrachloridocadmium(II) Fractional atomic coordinates and isotropic or equivalent isotropic displacement parameters (Å^2^)

**Table:**

	*x*	*y*	*z*	*U*_iso_*/*U*_eq_	
Cd1	0.55171 (2)	0.71231 (2)	0.76703 (2)	0.01848 (6)	
Cl1	0.46428 (8)	0.71615 (6)	0.59977 (5)	0.03304 (17)	
Cl2	0.63522 (6)	0.50197 (5)	0.84357 (5)	0.02149 (13)	
Cl3	0.37442 (6)	0.81359 (5)	0.89287 (5)	0.02465 (14)	
Cl4	0.73342 (7)	0.84425 (5)	0.73399 (5)	0.02646 (15)	
N1	0.2818 (2)	0.23733 (16)	0.94071 (16)	0.0168 (4)	
N2	0.1226 (2)	0.39279 (17)	0.88997 (16)	0.0181 (4)	
N3	0.1461 (2)	0.09936 (16)	0.83280 (16)	0.0187 (4)	
C1	0.1543 (2)	0.2704 (2)	0.90398 (19)	0.0169 (5)	
C2	0.2345 (3)	0.4354 (2)	0.9184 (2)	0.0196 (5)	
C3	0.3324 (3)	0.3361 (2)	0.95090 (19)	0.0188 (5)	
H3	0.420091	0.335580	0.976096	0.023*	
C4	0.3592 (3)	0.1135 (2)	0.9694 (2)	0.0238 (6)	
H4A	0.404112	0.081565	0.905313	0.036*	
H4B	0.292051	0.062933	1.008614	0.036*	
H4C	0.433169	0.113245	1.013424	0.036*	
C5	0.2286 (3)	0.5618 (2)	0.9053 (2)	0.0223 (6)	
H5	0.303810	0.592783	0.923318	0.027*	
C6	0.1138 (3)	0.6367 (2)	0.8666 (2)	0.0262 (6)	
H6	0.108142	0.721477	0.857448	0.031*	
C7	0.0009 (3)	0.5911 (2)	0.8392 (2)	0.0256 (6)	
H7	−0.079016	0.645928	0.812363	0.031*	
C8	0.0050 (3)	0.4727 (2)	0.8504 (2)	0.0251 (6)	
H8	−0.071002	0.443354	0.831666	0.030*	
C9	0.0679 (2)	0.1897 (2)	0.87987 (19)	0.0167 (5)	
C10	−0.0786 (3)	0.2061 (2)	0.9062 (2)	0.0232 (6)	
H10	−0.128823	0.270389	0.941296	0.028*	
C11	−0.1499 (3)	0.1252 (2)	0.8796 (2)	0.0284 (6)	
H11	−0.250913	0.134240	0.894811	0.034*	
C12	−0.0721 (3)	0.0318 (2)	0.8310 (2)	0.0297 (6)	
H12	−0.118633	−0.024474	0.811981	0.036*	
C13	0.0742 (3)	0.0213 (2)	0.8105 (2)	0.0242 (6)	
H13	0.127099	−0.044787	0.778788	0.029*	
N4	0.2726 (2)	0.08422 (17)	0.58031 (17)	0.0225 (5)	
N5	0.3173 (2)	0.23872 (17)	0.63174 (16)	0.0183 (4)	
N6	0.0130 (2)	0.34248 (19)	0.63388 (17)	0.0285 (5)	
C14	0.2197 (3)	0.2002 (2)	0.5887 (2)	0.0210 (5)	
C15	0.4022 (3)	0.0479 (2)	0.6177 (2)	0.0240 (6)	
H15	0.460532	−0.030079	0.620321	0.029*	
C16	0.4329 (3)	0.1443 (2)	0.65074 (19)	0.0203 (5)	
C17	0.2027 (3)	0.0022 (2)	0.5410 (2)	0.0327 (7)	
H17A	0.214666	0.017799	0.463828	0.049*	
H17B	0.100473	0.015804	0.567009	0.049*	
H17C	0.246445	−0.081565	0.566185	0.049*	
C18	0.5494 (3)	0.1647 (2)	0.6946 (2)	0.0262 (6)	
H18	0.628307	0.101148	0.709293	0.031*	
C19	0.5467 (3)	0.2756 (2)	0.7152 (2)	0.0275 (6)	
H19	0.624016	0.289983	0.745308	0.033*	
C20	0.4292 (3)	0.3712 (2)	0.6923 (2)	0.0258 (6)	
H20	0.430482	0.449280	0.705267	0.031*	
C21	0.3161 (3)	0.3527 (2)	0.6525 (2)	0.0217 (5)	
H21	0.237135	0.416540	0.638813	0.026*	
C22	0.0784 (3)	0.2703 (2)	0.5643 (2)	0.0222 (5)	
C23	0.0180 (3)	0.2607 (2)	0.4770 (2)	0.0317 (6)	
H23	0.069903	0.212218	0.427328	0.038*	
C24	−0.1193 (3)	0.3230 (3)	0.4639 (2)	0.0376 (7)	
H24	−0.164382	0.316423	0.405925	0.045*	
C25	−0.1891 (3)	0.3942 (3)	0.5354 (2)	0.0367 (7)	
H25	−0.284419	0.436304	0.529123	0.044*	
C26	−0.1179 (3)	0.4039 (3)	0.6174 (2)	0.0369 (7)	
H26	−0.165158	0.457220	0.664470	0.044*	

### Bis[2-methyl-3-(pyridin-2-yl)imidazo[1,5-a]pyridin-2-ium] tetrachloridocadmium(II) Atomic displacement parameters (Å^2^)

**Table:**

	*U*^11^	*U*^22^	*U*^33^	*U*^12^	*U*^13^	*U*^23^
Cd1	0.01851 (10)	0.01593 (10)	0.02228 (11)	−0.00275 (7)	−0.00489 (7)	−0.00489 (7)
Cl1	0.0463 (4)	0.0322 (4)	0.0245 (4)	−0.0092 (3)	−0.0153 (3)	−0.0035 (3)
Cl2	0.0204 (3)	0.0156 (3)	0.0287 (4)	−0.0012 (2)	−0.0057 (3)	−0.0044 (3)
Cl3	0.0189 (3)	0.0210 (3)	0.0350 (4)	0.0002 (2)	−0.0018 (3)	−0.0122 (3)
Cl4	0.0227 (3)	0.0189 (3)	0.0393 (4)	−0.0075 (2)	−0.0048 (3)	−0.0040 (3)
N1	0.0156 (11)	0.0138 (9)	0.0216 (11)	−0.0020 (8)	−0.0046 (8)	−0.0037 (8)
N2	0.0142 (10)	0.0168 (10)	0.0255 (12)	−0.0008 (8)	−0.0058 (9)	−0.0079 (9)
N3	0.0222 (11)	0.0131 (10)	0.0214 (12)	−0.0042 (8)	−0.0026 (9)	−0.0036 (9)
C1	0.0153 (13)	0.0165 (11)	0.0203 (13)	−0.0031 (9)	−0.0019 (10)	−0.0063 (10)
C2	0.0176 (13)	0.0195 (12)	0.0255 (14)	−0.0041 (10)	−0.0053 (10)	−0.0097 (11)
C3	0.0175 (13)	0.0157 (12)	0.0260 (14)	−0.0053 (10)	−0.0075 (10)	−0.0040 (11)
C4	0.0248 (14)	0.0130 (11)	0.0331 (16)	0.0000 (10)	−0.0101 (12)	−0.0011 (11)
C5	0.0265 (14)	0.0181 (12)	0.0253 (15)	−0.0093 (11)	−0.0030 (11)	−0.0051 (11)
C6	0.0342 (16)	0.0144 (12)	0.0304 (16)	−0.0003 (11)	−0.0064 (12)	−0.0068 (11)
C7	0.0240 (14)	0.0220 (13)	0.0285 (15)	0.0053 (11)	−0.0078 (11)	−0.0058 (11)
C8	0.0199 (14)	0.0255 (14)	0.0321 (16)	−0.0016 (11)	−0.0103 (11)	−0.0077 (12)
C9	0.0192 (13)	0.0172 (12)	0.0152 (13)	−0.0077 (10)	−0.0037 (10)	−0.0003 (10)
C10	0.0194 (14)	0.0278 (14)	0.0250 (15)	−0.0070 (11)	−0.0013 (11)	−0.0091 (12)
C11	0.0197 (14)	0.0370 (16)	0.0322 (16)	−0.0156 (12)	−0.0021 (12)	−0.0044 (13)
C12	0.0358 (17)	0.0301 (15)	0.0313 (16)	−0.0214 (13)	−0.0083 (13)	−0.0050 (13)
C13	0.0356 (16)	0.0161 (12)	0.0242 (15)	−0.0082 (11)	−0.0054 (12)	−0.0059 (11)
N4	0.0279 (12)	0.0167 (10)	0.0235 (12)	−0.0037 (9)	−0.0035 (9)	−0.0051 (9)
N5	0.0217 (11)	0.0153 (10)	0.0168 (11)	−0.0033 (8)	0.0008 (8)	−0.0021 (8)
N6	0.0257 (13)	0.0306 (12)	0.0241 (13)	0.0019 (10)	−0.0006 (10)	−0.0026 (10)
C14	0.0237 (14)	0.0206 (12)	0.0182 (14)	−0.0035 (10)	−0.0023 (10)	−0.0029 (11)
C15	0.0253 (15)	0.0188 (12)	0.0246 (15)	0.0017 (10)	−0.0019 (11)	−0.0031 (11)
C16	0.0236 (14)	0.0160 (12)	0.0184 (13)	−0.0007 (10)	0.0007 (10)	−0.0012 (10)
C17	0.0463 (18)	0.0232 (14)	0.0331 (17)	−0.0091 (12)	−0.0103 (14)	−0.0090 (12)
C18	0.0233 (14)	0.0266 (14)	0.0266 (15)	−0.0001 (11)	−0.0022 (11)	−0.0053 (12)
C19	0.0257 (15)	0.0318 (15)	0.0267 (15)	−0.0109 (12)	−0.0018 (11)	−0.0039 (12)
C20	0.0300 (15)	0.0211 (13)	0.0271 (15)	−0.0090 (11)	0.0031 (12)	−0.0066 (11)
C21	0.0259 (14)	0.0133 (12)	0.0236 (14)	−0.0019 (10)	0.0017 (11)	−0.0028 (10)
C22	0.0232 (14)	0.0192 (12)	0.0219 (14)	−0.0038 (10)	−0.0020 (11)	0.0010 (11)
C23	0.0366 (17)	0.0300 (15)	0.0280 (16)	−0.0036 (12)	−0.0081 (13)	−0.0033 (13)
C24	0.0360 (18)	0.0420 (17)	0.0339 (18)	−0.0085 (14)	−0.0133 (14)	0.0042 (14)
C25	0.0230 (15)	0.0453 (18)	0.0331 (18)	−0.0020 (13)	−0.0030 (13)	0.0095 (14)
C26	0.0288 (16)	0.0429 (17)	0.0296 (17)	0.0052 (13)	0.0033 (13)	−0.0018 (14)

### Bis[2-methyl-3-(pyridin-2-yl)imidazo[1,5-a]pyridin-2-ium] tetrachloridocadmium(II) Geometric parameters (Å, º)

**Table:**

Cd1—Cl1	2.4436 (7)	C12—C13	1.376 (4)
Cd1—Cl2	2.4448 (6)	C13—H13	0.9500
Cd1—Cl3	2.4496 (7)	N4—C14	1.343 (3)
Cd1—Cl4	2.4895 (6)	N4—C15	1.361 (3)
N1—C1	1.339 (3)	N4—C17	1.474 (3)
N1—C3	1.365 (3)	N5—C14	1.353 (3)
N1—C4	1.467 (3)	N5—C16	1.395 (3)
N2—C1	1.359 (3)	N5—C21	1.388 (3)
N2—C2	1.400 (3)	N6—C22	1.344 (3)
N2—C8	1.395 (3)	N6—C26	1.333 (3)
N3—C9	1.341 (3)	C14—C22	1.473 (3)
N3—C13	1.341 (3)	C15—H15	0.9500
C1—C9	1.475 (3)	C15—C16	1.367 (3)
C2—C3	1.360 (3)	C16—C18	1.414 (3)
C2—C5	1.421 (3)	C17—H17A	0.9800
C3—H3	0.9500	C17—H17B	0.9800
C4—H4A	0.9800	C17—H17C	0.9800
C4—H4B	0.9800	C18—H18	0.9500
C4—H4C	0.9800	C18—C19	1.350 (4)
C5—H5	0.9500	C19—H19	0.9500
C5—C6	1.348 (3)	C19—C20	1.424 (4)
C6—H6	0.9500	C20—H20	0.9500
C6—C7	1.421 (4)	C20—C21	1.347 (3)
C7—H7	0.9500	C21—H21	0.9500
C7—C8	1.334 (3)	C22—C23	1.387 (4)
C8—H8	0.9500	C23—H23	0.9500
C9—C10	1.381 (3)	C23—C24	1.382 (4)
C10—H10	0.9500	C24—H24	0.9500
C10—C11	1.389 (3)	C24—C25	1.364 (4)
C11—H11	0.9500	C25—H25	0.9500
C11—C12	1.377 (4)	C25—C26	1.389 (4)
C12—H12	0.9500	C26—H26	0.9500
			
Cl1—Cd1—Cl2	108.30 (2)	N3—C13—C12	123.6 (2)
Cl1—Cd1—Cl3	113.98 (2)	N3—C13—H13	118.2
Cl1—Cd1—Cl4	108.24 (2)	C12—C13—H13	118.2
Cl2—Cd1—Cl3	110.40 (2)	C14—N4—C15	110.5 (2)
Cl2—Cd1—Cl4	115.56 (2)	C14—N4—C17	126.9 (2)
Cl3—Cd1—Cl4	100.36 (2)	C15—N4—C17	122.6 (2)
C1—N1—C3	110.67 (19)	C14—N5—C16	109.32 (19)
C1—N1—C4	126.46 (19)	C14—N5—C21	129.3 (2)
C3—N1—C4	122.86 (19)	C21—N5—C16	121.3 (2)
C1—N2—C2	109.13 (19)	C26—N6—C22	116.7 (2)
C1—N2—C8	130.01 (19)	N4—C14—N5	106.7 (2)
C8—N2—C2	120.81 (19)	N4—C14—C22	127.3 (2)
C9—N3—C13	116.4 (2)	N5—C14—C22	125.9 (2)
N1—C1—N2	106.48 (18)	N4—C15—H15	126.3
N1—C1—C9	126.6 (2)	N4—C15—C16	107.5 (2)
N2—C1—C9	126.9 (2)	C16—C15—H15	126.3
N2—C2—C5	119.3 (2)	N5—C16—C18	119.2 (2)
C3—C2—N2	106.19 (19)	C15—C16—N5	106.1 (2)
C3—C2—C5	134.5 (2)	C15—C16—C18	134.8 (2)
N1—C3—H3	126.2	N4—C17—H17A	109.5
C2—C3—N1	107.5 (2)	N4—C17—H17B	109.5
C2—C3—H3	126.2	N4—C17—H17C	109.5
N1—C4—H4A	109.5	H17A—C17—H17B	109.5
N1—C4—H4B	109.5	H17A—C17—H17C	109.5
N1—C4—H4C	109.5	H17B—C17—H17C	109.5
H4A—C4—H4B	109.5	C16—C18—H18	120.5
H4A—C4—H4C	109.5	C19—C18—C16	119.1 (2)
H4B—C4—H4C	109.5	C19—C18—H18	120.5
C2—C5—H5	120.8	C18—C19—H19	119.7
C6—C5—C2	118.5 (2)	C18—C19—C20	120.6 (2)
C6—C5—H5	120.8	C20—C19—H19	119.7
C5—C6—H6	119.5	C19—C20—H20	119.4
C5—C6—C7	121.1 (2)	C21—C20—C19	121.1 (2)
C7—C6—H6	119.5	C21—C20—H20	119.4
C6—C7—H7	119.4	N5—C21—H21	120.6
C8—C7—C6	121.3 (2)	C20—C21—N5	118.7 (2)
C8—C7—H7	119.4	C20—C21—H21	120.6
N2—C8—H8	120.5	N6—C22—C14	114.6 (2)
C7—C8—N2	119.0 (2)	N6—C22—C23	123.2 (2)
C7—C8—H8	120.5	C23—C22—C14	122.3 (2)
N3—C9—C1	113.4 (2)	C22—C23—H23	120.7
N3—C9—C10	124.4 (2)	C24—C23—C22	118.6 (3)
C10—C9—C1	122.2 (2)	C24—C23—H23	120.7
C9—C10—H10	121.2	C23—C24—H24	120.5
C9—C10—C11	117.6 (2)	C25—C24—C23	119.1 (3)
C11—C10—H10	121.2	C25—C24—H24	120.5
C10—C11—H11	120.4	C24—C25—H25	120.7
C12—C11—C10	119.1 (2)	C24—C25—C26	118.7 (3)
C12—C11—H11	120.4	C26—C25—H25	120.7
C11—C12—H12	120.6	N6—C26—C25	123.7 (3)
C13—C12—C11	118.9 (2)	N6—C26—H26	118.1
C13—C12—H12	120.6	C25—C26—H26	118.1
			
N1—C1—C9—N3	−40.5 (3)	N4—C14—C22—N6	138.5 (3)
N1—C1—C9—C10	138.2 (3)	N4—C14—C22—C23	−40.7 (4)
N2—C1—C9—N3	137.1 (2)	N4—C15—C16—N5	0.0 (3)
N2—C1—C9—C10	−44.3 (4)	N4—C15—C16—C18	−178.8 (3)
N2—C2—C3—N1	0.9 (3)	N5—C14—C22—N6	−36.2 (4)
N2—C2—C5—C6	0.7 (4)	N5—C14—C22—C23	144.6 (3)
N3—C9—C10—C11	−1.7 (4)	N5—C16—C18—C19	−1.1 (4)
C1—N1—C3—C2	−0.7 (3)	N6—C22—C23—C24	−3.7 (4)
C1—N2—C2—C3	−0.8 (3)	C14—N4—C15—C16	−0.2 (3)
C1—N2—C2—C5	176.8 (2)	C14—N5—C16—C15	0.1 (3)
C1—N2—C8—C7	−176.7 (3)	C14—N5—C16—C18	179.2 (2)
C1—C9—C10—C11	179.8 (2)	C14—N5—C21—C20	−177.3 (2)
C2—N2—C1—N1	0.4 (3)	C14—C22—C23—C24	175.4 (2)
C2—N2—C1—C9	−177.6 (2)	C15—N4—C14—N5	0.2 (3)
C2—N2—C8—C7	0.5 (4)	C15—N4—C14—C22	−175.3 (2)
C2—C5—C6—C7	−0.1 (4)	C15—C16—C18—C19	177.6 (3)
C3—N1—C1—N2	0.2 (3)	C16—N5—C14—N4	−0.2 (3)
C3—N1—C1—C9	178.2 (2)	C16—N5—C14—C22	175.4 (2)
C3—C2—C5—C6	177.5 (3)	C16—N5—C21—C20	−0.4 (4)
C4—N1—C1—N2	178.9 (2)	C16—C18—C19—C20	−0.6 (4)
C4—N1—C1—C9	−3.1 (4)	C17—N4—C14—N5	177.9 (2)
C4—N1—C3—C2	−179.5 (2)	C17—N4—C14—C22	2.3 (4)
C5—C2—C3—N1	−176.2 (3)	C17—N4—C15—C16	−177.9 (2)
C5—C6—C7—C8	−0.3 (4)	C18—C19—C20—C21	2.0 (4)
C6—C7—C8—N2	0.1 (4)	C19—C20—C21—N5	−1.4 (4)
C8—N2—C1—N1	177.8 (2)	C21—N5—C14—N4	177.0 (2)
C8—N2—C1—C9	−0.2 (4)	C21—N5—C14—C22	−7.4 (4)
C8—N2—C2—C3	−178.5 (2)	C21—N5—C16—C15	−177.4 (2)
C8—N2—C2—C5	−0.9 (4)	C21—N5—C16—C18	1.7 (3)
C9—N3—C13—C12	1.6 (4)	C22—N6—C26—C25	1.8 (4)
C9—C10—C11—C12	1.5 (4)	C22—C23—C24—C25	1.7 (4)
C10—C11—C12—C13	0.2 (4)	C23—C24—C25—C26	1.7 (4)
C11—C12—C13—N3	−1.8 (4)	C24—C25—C26—N6	−3.6 (5)
C13—N3—C9—C1	178.8 (2)	C26—N6—C22—C14	−177.2 (2)
C13—N3—C9—C10	0.2 (4)	C26—N6—C22—C23	1.9 (4)

### Bis[2-methyl-3-(pyridin-2-yl)imidazo[1,5-a]pyridin-2-ium] tetrachloridocadmium(II) Hydrogen-bond geometry (Å, º)

**Table:**

*D*—H···*A*	*D*—H	H···*A*	*D*···*A*	*D*—H···*A*
C4—H4*C*···Cl3^i^	0.98	2.72	3.664 (3)	162
C5—H5···Cl3	0.95	2.71	3.446 (2)	135
C7—H7···Cl4^ii^	0.95	2.72	3.651 (3)	166
C8—H8···Cl2^ii^	0.95	2.75	3.518 (3)	138
C12—H12···Cl4^iii^	0.95	2.70	3.644 (2)	171
C17—H17*A*···Cl4^iv^	0.98	2.79	3.695 (3)	155
C17—H17*C*···Cl1^v^	0.98	2.80	3.721 (3)	158
C23—H23···Cl4^iv^	0.95	2.69	3.576 (3)	156
C26—H26···Cl2^ii^	0.95	2.84	3.689 (3)	149

Symmetry codes: (i) −*x*+1, −*y*+1, −*z*+2; (ii) *x*−1, *y*, *z*; (iii) *x*−1, *y*−1, *z*; (iv) −*x*+1, −*y*+1, −*z*+1; (v) *x*, *y*−1, *z*.
